# Functional outcomes of otosclerosis surgery

**DOI:** 10.1016/j.bjorl.2025.101679

**Published:** 2025-07-11

**Authors:** Nadia Romdhane, Dorra Chiboub, Amal Nessib, Ameni Amri, Ines Hariga, Chiraz Chaouch Mbarek

**Affiliations:** University Tunis El Manar, Faculty of Medicine of Tunis, Habib Thameur Hospital, Ears Nose and Throat, Head and Neck Surgery Department, Tunis, Tunisia

**Keywords:** Otosclerosis, Surgery, Results, Deafness

## Abstract

**Objectives:**

The objectives of this work were to evaluate the results of otosclerosis surgery and to study the predictors of its functional success.

**Methods:**

Our study was retrospective on 322 patients, equivalent to 418 ears operated in the otolaryngology and head and neck surgery department, from January 2000 to December 2020.

**Results:**

Hearing success was noted in 89.5% of patients considering the Postoperative Residual Air Bone Gap (PRABG) ≤10 dB criterion; in 94.3% of patients according to the conductive gain ≥20 dB criterion; in 96.4% of patients based on the improvement of cochlear reserve criterion; in 88.8% according to the Air Bone Gap improvement (ABGi) ≥70% criterion and in 81.8% considering all these criteria. The short, medium and long-term postoperative tonal audiometries could be superimposed, reflecting the stability of the hearing results. A regression of tinnitus has been reported in 79.2% of cases. The independent predictors of hearing success were the Aubry's audiometric stages I and II, mean preoperative air curve >40 dB and platinotomy. The independent predictors of tinnitus regression retained were male sex, no exposure to loud noise and Veillon radiological stages I and II.

**Conclusion:**

The identification of factors influencing the functional results of surgery allows a better selection of candidates for the intervention and more relevant information for patients.

**Level of evidence:**

Level 3: Non-randomized controlled cohort or follow-up study.

## Highlights

• Better results with early Aubry stages (I and II).
• Better functional outcomes with early Portmann stages.
• Stapedectomy exposes to a higher risk of air bone gap reopening in long-term.
• Better auditory results and less complications with calibrated platinotomy.

## Introduction

Otosclerosis is a primary osteodystrophy of the otic capsule due to abnormal bone turnover resulting in stapedo-vestibular ankylosis and causing conductive hearing loss. Its pathogenesis remains hypothetical. Treatment is essentially surgical. Stapes surgery is a functional surgery with generally excellent results. The functional outcome depends on patient-related factors and perioperative data. Auditory results differ from one patient to another and depend on a good therapeutic indication, preoperative audiometric stage, duration of evolvement, radiological stage, surgical technique and intraoperative findings.

Identifying predictive factors of the surgical result allows a better selection of candidates for surgery, a good planning of intervention, and a more relevant information of the patients concerning the expected outcomes. Our aim is to study predictive factors of clinical and audiometric success of otosclerosis surgery.

## Methods

Retrospective study on patients operated for otosclerosis during a 21-year period (January 2000 ‒ December 2020), with a minimum follow-up of 2-years. All surgeons were experienced seniors. The diagnosis was evoked by clinical, audiometric and radiological data, and confirmed by intraoperative stapedo-vestibular ankylosis. Patients with hearing aids or without surgical indication were not included. Incomplete records were excluded, especially without postoperative audiometry. Surgical indication was determined considering the type of hearing loss and the patient's choice. In case of “pure” conductive deafness, we operated on patients with an Air Bone Gap (ABG) ≥ 30 dB. In cases of mixed deafness with a lowered Bone Conduction (BC) of more than 50 dB with intelligibility problems, given the low chances of recovery, hearing aids were indicated. The American Academy of Otolaryngology, Head and Neck surgery (AAO-HNS) recommendations of 1995 were applied.^1^^,^^2^ The average Air Conduction threshold (AC) is the average AC over the frequencies 500, 1000, 2000, and 3000 Hz. The average bone Conduction threshold (BC) is the mean BC at 500, 1000, 2000 and 3000 Hz. Success criteria were defined by postoperative Air Bone Gap (PABG) which is the difference between average postoperative AC and BC thresholds. Surgical success was defined by a PABG ≤ 10 dB. Air Conduction Gain (AG) is the difference between average preoperative and postoperative AC thresholds. Surgical success was also defined by a transmission gain value ≥20 dB. Bone Gain (BG), or Study of cochlear reserve, is the difference between pre- and postoperative average BC thresholds. A negative value indicated an improvement in bone conduction, thus a successful outcome; a zero-value indicated that bone conduction was maintained; and a positive value indicated that bone conduction was worsened. Air Bone Gap improvement (ABGi), or Relative Gain, is defined as the percentage improvement in the postoperative ABG compared with the preoperative ABG. An ABGi ≥ 70% indicated success. Surgical success has been considered when all the four main end points were achieved (PABG ≤ 10 dB, transmission gain value ≥ 20 dB, Bone Gain, ABGi ≥ 70%).

## Results

During the 21-year study period (2000–2020), 484 ears were operated. The frequency of surgery was 24.2 ears per year. After applying the exclusion criteria, we selected 418 ears. We enrolled 322 patients, of whom almost a third (29.8%) underwent bilateral surgery. A female predominance was noted, 215 women (66.8%) for 107 men, with a sex-ratio of 0.5. The mean age of surgery was 39.4-years. The onset was progressive in all patients. The average age of onset of hypoacusis was 34.5-years. The evolution ranged from a few months to 20-years, with a mean progression before surgery of 4.7-years. Hypoacusis was the main symptom, present in all patients. Bilaterality was noted in 67.7% of cases. Hearing loss was isolated in 23.6% of cases. Tinnitus was reported by 76.4% of patients. It was bilateral in 154 patients (47.8%). Vertigo was noted in 6.5% of patients, and Willis paracusis in 10.9%. Preoperative tonal audiometry revealed conductive hearing loss in 73.7% of cases, and mixed hearing loss in 26.3%. The deafness was bilateral in 62.7% of cases. The average preoperative ABG was 34.1 dB. The average AC threshold was 49.9 dB, and the average BC threshold was 15.8 dB. The bone curve was decreased by more than 20 dB in 48.3% of cases. The distribution of audiograms according to Aubry's classification divided our population into 4 groups:

Stage I (pure conductive hearing loss with or without Carhart effect): 50.9% of cases;

Stage II (BC no longer rises after 2000 Hz): 23.7%;

Stage III (BC with a deficit of over 30 dB at 1000 and/or 2000 Hz): 21.8%;

Stage IV (BC greater than 40 dB at 1000 Hz with a drop in high frequencies above 4000 Hz): 3.6%.

Vocal audiometry was performed in 345 patients (82.5%). The curve was right shifted in all cases. The mean intelligibility threshold was 51 dB, with extremes ranging from 18 dB to 90 dB. The intelligibility threshold was consistent with the mean air conduction threshold of 49.9 dB. The maximum intelligibility and discrimination percentage were 100% in all cases.

**Fig. 1:**
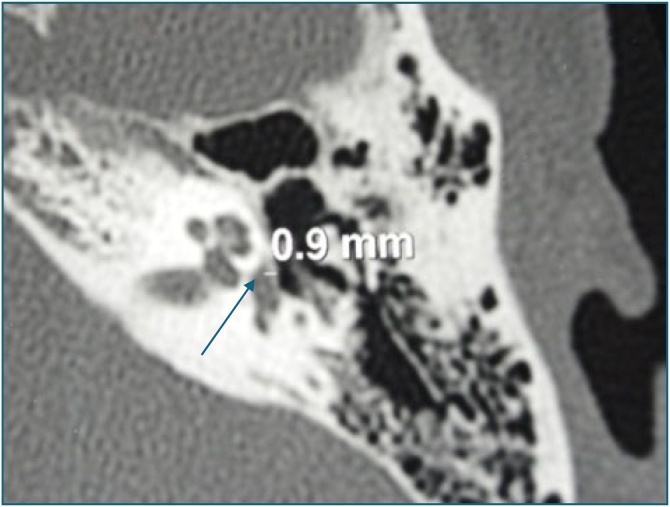
Temporal bone axial CT scan: prestapedial hypodensity of 0.9 mm.

A Computed Tomography (CT) scan of the temporal bone was performed in 76.5% of cases. It was normal in 12.5% of cases and had a sensitivity for detecting otosclerotis of 87.5% (Fig. 1).

**Fig. 2:**
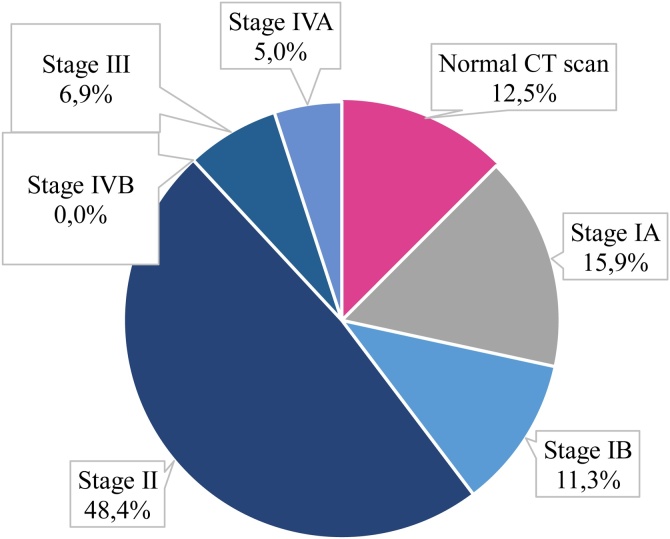
Veillon's radiological stages.

Scannographic Veillon Classification was adopted. Most ears (48.4%) were classified as stage II. The different stages are illustrated in Fig. 2.

Ninety-six patients (29.8%) underwent bilateral surgery, with a mean time between surgeries of 2.3-years. All cases were operated under general anaesthesia, via transmeatic approach under a microscope. The stapes was fixed in all patients. Platinal involvement enabled otosclerosis to be classified into 5 stages according to Portmann’s classification. Most ears (94.4%) had platinal involvement of stage II (25.8%), III (39.2%) or IV (29.4%). Stage I and V were noted in respectively 3.7% and 1.9% of cases. Involvement of the round window was noted in 11 cases (2.6%).

In 80 ears (19.1), we encountered intraoperative difficulties. The most frequent variant (10.8%) was procidence of the bony canal of the facial nerve next to the oval window, leading to abstention from platinal procedure in 4 cases. The Descending Branch of the Incus (DBI) was pathological in 33 cases. It was too small in 12 cases and lysed in 21. The cases of significant lysis of the DBI led to fixing the piston at the malleus in 2 cases, and to abstain in 4 patients in view of a later procedure consisting in placing a malleus piston, unavailable intraoperatively. Fixity of the malleus was noted in 3 patients and repaired in all cases, its head was removed and replaced by a cartilage graft placed over the incus and the piston. Two cases of otosclerosis obliterans were associated with an involvement of the round window, and a platinotomy was performed without incident.

The surgical techniques varied depending on the available equipment; for example, laser was used whenever it was available, but this was not always the case. The platinum procedures performed were calibrated platinotomy (77.5%), partial platinectomy (11%) and total platinectomy (9.6%). A platinum procedure was performed in 410 ears. Abstention was preferred in 8 patients (1.9%) due to extensive lysis of the DBI (4 cases) and a procident facial canal (4 cases). Platinotomy was performed with a perforator in 84.9% of cases, with a diode laser in 9.6% and with a Skeeter in 5.5%. The piston was made of Teflon in all cases. Prosthesis diameters were 0.4 mm in 48.9% of cases and 0.6 mm in 51.1%. Its length was identified by measuring the distance between the DBI and the footplate of the stapes. It was 4.25 mm in 15.3% of cases, 4.50 mm in 58.5% and 4.75 mm in 26.2%. Interposition or filling material was recommended in 71% of cases, mostly using fat from the ear lobule (98.6%). The graft interposition between the oval window and the piston was systematic in case of platinectomy. Using graft around the piston was performed on a case-by-case basis in front of a calibrated platinotomy, to seal a possible breach. This procedure was performed in 65.1% cases of platinotomy. A platinal fissure during platinotomy occurred in 77 cases (18.4%), requiring partial (46 cases) or total platinectomy (31 cases).

When it comes to perioperative complications, 47 operations (11.3%) resulted in another intraoperative incident. Sectioning or stretching of the tympanic cord was the most frequent. It required partial (46 cases) or total platinectomy (31 cases). Second most frequent incident was tympano-meatal flap injury in 14 cases (3.3%). Tympanic tear occurred in 4 cases (1%), followed by floating plate in 8 cases (2%). The repair of a tympano-meatal flap tear required the use of cartilage or superficial temporal fascia graft in 3 cases, fat plugging in 12 cases, and simple approximation of edges in 3 cases.

As for postoperative complications, moderate vertigo was observed in 146 patients (34.9%), 21 of whom had preexisting vertigo. Vestibular examinations conducted on these patients showed no abnormalities. The vertigo resolved within an average of 2-days after surgery for 77 patients. However, 69 patients experienced prolonged vertigo, which resolved after an average of 3-months. Other complications included one case of peripheral facial paralysis, presenting as homolateral facial paresis homolateral to the operated ear, which occurred immediately after surgery and resolved during follow-up. Among the 21 cases of stretching or sectioning of the tympanic cord, 11 patients (52.4% of cord injuries and 2.6% of operated ears) experienced dysgeusia, which did not improve throughout the follow-up period. One patient reported tongue hypoesthesia. Additionally, a tympanic perforation not detected during surgery was discovered postoperatively in one patient. The perforation was a posterior arcuate non-marginal type and required a subsequent type I tympanoplasty.

**Fig. 3:**
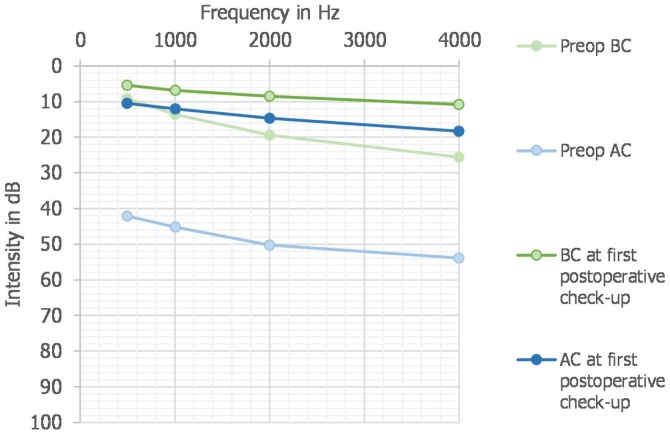
Audiograms preoperatively and at first postoperative control.

Postoperative audiometric assessments were scheduled at 1-, 3-, and 12-months. However, in practice, patients may miss their appointments, and scheduling depends on availability. The mean audiometric ABG was 4.8 dB. The mean AC was 13.4 dB, and the mean BC was 8.6 dB (Fig. 3).

Audiometric testing was carried out regularly. The last audiometry was performed on average 30.1-months postoperatively, with extremes ranging from one to 240-months. By comparing the audiometric data of the same patients at the first audiometric test, at one year after surgery and at the last test, we obtained superimposable curves in all cases. The mean PABG was 4.8 dB with extremes ranging from 0 to 30 dB. A PABG ≤ 10 dB, defining an excellent hearing result, was found in 89.5% of ears. ABG closure with a PABG between 11 and 20 dB, defining a good functional result, was noted in 98.1%.

The mean AG was 36.5 dB, with extremes ranging from -11 to 86 dB. Satisfactory AG (≥ 20 dB) was achieved in 94.3% of ears.

The mean BG was -7.1 dB, ranging from -69 to 28 dB. Cochlear reserve deteriorated in 3.6%, remained unchanged in 27.3% and improved in 69.1% of ears. Surgery was considered successful if BG improved or remained unchanged, which was the case in 96.4% of patients.

The average ABGi was 87.3%. Considering the parameter ABGi ≥70%, the success rate was 88.8%.

The percentage of patients with simultaneous success in all four parameters was 81.8%.

A uni and multivariate analytical study were conducted to identify epidemiological, clinical, paraclinical and therapeutic factors with significant impact on hearing outcomes.

The factors influencing hearing outcome were:

• Duration of evolution <1-year (p = 0.019);
• Absence of otitic history (p = 0.041);
• Preserved bone curve (p = 0.048);
• Presence of a Carhart notch (p = 0.004);
• Aubry stages I and II (p = 0.031);
• Preoperative air conduction >40 dB (p = 0.029);
• Absence of anatomical variants CT (p = 0.047);
• Calibrated platinotomy (p = 0.022).

To identify factors directly related to success, and to eliminate variables that might distort our study, we conducted a multivariate study using logistic regression. We included the factors retained in the univariate study.

Four factors were retained as independent predictive of success (Table 1):

• Aubry stages I and II;
• Preoperative mean air conduction >40 Db;
• Calibrated platinotomy.

**Table 1:** Multivariate analytical study of independent factors predictive of success.

Factors retained	B	Confidence interval	p	Odds Ratio
Aubry stages I and II	1.150	[0.107; 0.937]	**0.038**	0.317
Preoperative mean air conduction > 40 dB	1.616	[1.545; 16.411]	**0.007**	5.035
Calibrated platinotomy	1.926	[0.027; 0.795]	**0.026**	0.146

## Discussion

Otosclerosis is a frequent cause of deafness in adults, with a prevalence ranging from 0.3% to 2%.^3^^,^^4^

The average age of onset ranges from 15- to 45-years, with a peak in the thirties.^3^ The prevalence is twice as high in women as in men, with a sex-ratio of 0.5.^3^^,^^5^

Stapedo-vestibular ankylosis leads to conductive hearing loss.^6^^,^^7^ Exploration of stapedial reflexes, absent in established otosclerosis, is an integral part of the audiometric testing.

A CT scan of the temporal bone can detect thickening of the platinum, with or without prespace hypodensity. A normal CT scan does not exclude the diagnosis.^4^^,^^8^

Surgery is the treatment of choice. A piston is inserted to restore the connection between the ossicular chain and the oval window.^4^^,^^9^

Most authors perform the first postoperative audiometry at 3-months, then one year post-operatively.^3^^,^^10^

Preoperative audiometry is ideally performed one month before surgery, at most 3-months.^3^

Although early audiometric results do not reflect the definitive outcome, they allow assessing auditory outcome, detecting complications and comparing surgical techniques.

The Swedish national study on stapes surgery highlights that one-year outcomes provide a more objective assessment of auditory improvement, aligning with AAO-HNS recommendations, and showing a successful ABG closure to ≤10 dB in 69% of cases.^11^

Despite variability in timing of tone audiometry and the diversity of criteria for evaluating results, our functional success rates remain comparable to literature (Table 2).

**Table 2:** Early functional success rates in literature.

Series	Number of ears	Evaluation methods	Success rates (%)
Stromback ^11^	1688	PABG ≤ 10 dB	69
		AG ≥ 20 dB	63
		BG < 5 dB	93
Vincent ^12^	3050	PABG ≤ 10 dB	94.2
		ABGi ≥ 70%	85.5
Crompton ^3^	657	PABG ≤ 10 dB	88
		AG ≥ 20 dB	75
		BG < 5 dB	86
Kishimoto ^10^	234	PABG ≤ 10 dB	77.58
Odat ^13^	58	PABG ≤ 10 dB	75.9
		PABG ≤ 20 dB	89.7
Our serie	418	PABG ≤ 10 dB	89.5
		AG ≥ 20 dB	94.3
		BG ≤ 0 dB	96.4
		ABGi ≥ 70%	88.8

Results at one year or more provide, according to the AAO-HNS, a more objective assessment of the final outcome than early results.^2^ Success rates vary from series to series but are generally satisfactory (Table 3).

**Table 3:** Functional success rate at one-year post-op in literature.

Series	Number of ears	Evaluation methods	Success rates (%)
Stromback ^11^	1688	PABG ≤ 10 dB	68
		AG ≥ 20 dB	63
		BG ≤ 0 dB	82
Vincent ^12^	3050	PABG ≤ 10 dB	95.6
Khorsandi ^14^	995	PABG ≤ 10 dB	93.6
		AG ≥ 10db	
		BG ≤ 0 dB	
Rajput ^5^	48	PABG ≤ 10 dB	70.8
Our serie	418	PABG ≤ 10 dB	89.5
		AG ≥ 20 Db	94.3
		BG ≤ 0 dB	96.4
		ABGi ≥ 70%	88.8

In our study, early and late functional results are superimposable.

Very few studies have a follow-up exceeding one year. Most authors agree on the stability and durability of results, like our study.^12^, ^13^, ^14^, ^15^

Many authors studied the influence of epidemiological, clinical, audiometric, radiological and peroperative factors on the audiometric results, however, no consensus was established.

Some authors report a significant improvement in PABG and BG in patients under 35-years-old.^10^ Others found that age greater than 40 is often associated with good functional results, particularly with more frequent ABG closure.^16^

A symptom progression shorter than one year would be associated with better ABG closure in short-term,^17^ but medium- and long-term results remain comparable to patients with longer delays. In our series, a delay of evolution < 1-year was predictive of better ABG closure and higher conductive gain and ABGi.

Wegner et al. examined the correlation between CT findings and surgical outcomes in otosclerosis, highlighting that extensive otosclerotic foci, cochlear involvement, and round window obliteration were associated with lower surgical success rates and increased complications. These findings align with the present study.^18^

Aubry stages I and II have been associated in most series with better short- and long-term functional results, for both air- and bone-conduction gain.^11^

Recent studies affirmed that stapedial surgery in ears with large or reduced ABG has comparable audiometric results, and is equally subject to post-operative complications, sensorineural hearing loss and vertigo.^19^^,^^20^ Toscano and Kishimoto recommend surgery when the preoperative ABG is higher than or equal to 20 dB.^9^^,^^10^

For advanced Aubrey stages, stapes surgery can be a safe and effective option, providing significant improvement in hearing and speech perception while reducing the need for cochlear implantation. In a study where all patients were initially candidates for cochlear implants, stapes surgery resulted in an average pure-tone air conduction gain of 33 dB and a mean speech perception improvement of 54.4%, with 87% of patients achieving sufficient hearing restoration to no longer require implantation. Given its lower cost, reduced invasiveness, and minimal need for rehabilitation compared to cochlear implants, stapes surgery should be considered a first-line treatment before resorting to cochlear implantation.^21^ However, if stapedotomy fails, cochlear implantation remains a highly effective alternative, offering superior speech recognition and stable long-term outcomes. Studies show that while initial speech perception is low pre-implantation, cochlear implant recipients experience substantial gains, with scores reaching 72% in sentence recognition tests after three years, comparable to post-lingual cochlear implant patients with other etiologies.^22^^,^^23^

The lesion state of the platinum reflects the extent of otosclerosis. Early stages with minimal involvement have better prognosis.^12^ Indeed, Portmann stage I is correlated with better functional results. In our study, Portmann stages I, II and III were associated with better hearing results, without being significant.

There is no difference between stapedotomy and stapedectomy in terms of ABG closure and stability, according to some authors^9^^,^^14^ (Table 4). According to others, stapedectomy exposes to a higher risk of ABG reopening and labyrinthization in long-term.^24^

**Table 4:** Auditory success according to platinary gesture in literature.

Series	Percentage of patients with PABG ≤ 10 dB	
	Platinotomy	Platinectomy
Dubreuil ^20^	80 %	85 %
Khorsandi ^14^	94,2 %	93,4 %
Our serie	93,9 %	86,7 %

In our study, platinotomy was associated with better closure of ABG and AG. It was considered an independent predictive factor of hearing success.

The nature of interposition tissue depends on the surgeon's habits. Several authors have studied audiometric results in relation with interposition material used, comparing hyaluronic acid gel, Geofoam, fat or vein graft. No significant correlation was established, and auditory results do not appear to be influenced by the nature of the graft.^25^^,^^26^

Most authors concluded there was no superiority of one piston over another, and the surgeon's experience in choosing measurements was the predominant factor.^27^

The piston’s length should be adapted for each patient by measuring the distance between the DBI and the plate.^9^^,^^28^ Ideally, this measurement leads to a prosthesis extending into the vestibule by 0.25 to 0.5 mm.^28^ Some otologists use standardized lengths, which is a cause of surgical failure. In fact, a series studying revision surgeries showed that inadequate prosthesis length was the cause of 73.5% of functional failures.^29^ Blijleven et al. found that using a 0.6-mm piston resulted in significantly better audiometric outcomes compared to a 0.4-mm piston, particularly in air conduction gain, ABG closure, and speech reception. Importantly, the larger piston size did not lead to a higher incidence of complications or revision surgeries.^30^ Blijleven et al. also evaluated the outcomes of revision stapes surgery in otosclerosis patients and found that while the success rates were lower than in primary stapes surgery, significant improvements in hearing were still achieved. The mean ABG improved by 19 dB, with 80% of cases achieving an ABG closure to 20 dB or less, and 38% achieving closure to 10 dB or less. Additionally, no cases of profound sensorineural hearing loss were observed, confirming that revision surgery remains a valuable and safe option for patients with persistent or recurrent conductive hearing loss.^31^

Nowadays, outcomes are no longer solely defined by audiometric measures but also by their impact on quality of life. Lailach et al. conducted a two-phase study to assess Health-Related Quality of Life (HRQoL) in otosclerosis patients undergoing stapes surgery. The first phase focused on the development and validation of the Stapesplasty Outcome Test-25 (SPOT-25), a disease-specific tool designed to capture the broader impact of otosclerosis beyond audiometric outcomes. This validation study confirmed that SPOT-25 is a reliable and valid measure of HRQoL, addressing key aspects such as hearing function, tinnitus, mental well-being, and social restrictions. In the second phase, the authors applied this newly validated instrument in a clinical study to evaluate HRQoL changes before and after stapes surgery. Their findings revealed that while audiometric improvements correlated well with the “hearing function” domain, they did not fully reflect patient experiences in psychosocial aspects such as tinnitus distress, emotional well-being, and social engagement. The study further demonstrated that all patients reported some level of benefit postoperatively, as measured by the Glasgow Benefit Inventory (GBI). These results underscore the limitations of relying solely on traditional hearing tests and highlight the importance of integrating HRQoL assessments into routine clinical practice to provide a more comprehensive evaluation of surgical success.^32^^,^^33^ Blijleven et al. highlighted that while postoperative improvements in ABG are often used to assess surgical success, these objective measures do not always align with patients' subjective experiences. Their study validated the Stapesplasty Outcome Test-25 (SPOT-25) as a disease-specific tool for evaluating Health-Related Quality of Life (HRQoL) in otosclerosis patients undergoing primary stapes surgery. They found that postoperative quality of life is influenced by factors beyond hearing thresholds, including tinnitus perception, mental well-being, and social restrictions.^34^ In our current study, integrating disease-specific HRQoL assessments like SPOT-25 would have provided a more comprehensive evaluation beyond audiometric outcomes. Future publications should incorporate validated patient-reported outcome measures to better capture the broader impact of stapes surgery on the quality of life.

This study has several limitations that should be acknowledged. Firstly, its retrospective nature limits the ability to control for confounding variables and may introduce biases in data collection and interpretation. Additionally, incomplete pre- and postoperative assessments, particularly in long-term follow-ups, may have affected the accuracy of the results, as some patients missed scheduled audiometric evaluations. Selection biases could also be present, as the study excluded patients with hearing aids or those without surgical indications, potentially skewing the population toward those with more favorable outcomes. Furthermore, the involvement of different surgeons and the use of varying surgical techniques, such as the inconsistent availability of laser equipment, may have introduced variability in outcomes. Lastly, tinnitus, a common postoperative complaint, was assessed subjectively and not through standardized, measurable metrics, which limits the ability to objectively evaluate its impact on patient outcomes. Future research on otosclerosis outcomes should incorporate gustatory assessments like the Taste Strip Test and electrogustometry to evaluate potential nerve-related taste disturbances post-stapedotomy. Additionally, food preference and quality-of-life questionnaires can help quantify long-term sensory and dietary changes in affected patients. Integrating these tests into otosclerosis studies may provide insights into nerve preservation strategies and patient-centered surgical approaches.^35^

## Conclusions

Identifying predictive factors of surgical outcome enables better selection of candidates, better planning, and more pertinent information for patients.

The impact of these parameters varies from author to author, due to the disparity of evaluation criteria and the absence of universal standardized recommendations. It would be useful to recommend evaluation parameters based on scientific evidence.

## ORCID ID

Nadia Romdhane: 0000-0003-1925-4441

Dorra Chiboub: 0000-0001-5915-9954

Amal Nessib: 0009-0000-1261-3267

Ines Hariga: 0009-0002-7940-4632

Chiraz Chaouch Mbarek: 0009-0006-1858-6240

## Ethics approval and informed consent

Due to its retrospective and observational nature, this work was not subject to the prior consent of the patients included in it.

## Funding

This research did not receive any specific grant from funding agencies in the public, commercial, or not-for-profit sectors.

## Declaration of competing interest

The authors declare no potential conflicts of interest with respect to the research, authorship, and/or publication of this article. There are no financial nor personal relationships with other people or organizations that could inappropriately influence this work.
